# An integrated smoking cessation and alcohol intervention among Hong Kong Chinese young people: Study protocol for a feasibility randomized controlled trial

**DOI:** 10.1371/journal.pone.0289633

**Published:** 2023-08-03

**Authors:** Ka Yan Ho, Katherine Ka Wai Lam, Cynthia Wu, Doris Y. P. Leung, Getaneh Mulualem Belay, Qi Liu, Yim Wah Mak

**Affiliations:** School of Nursing, Hong Kong Polytechnic University, HKSAR, Hong Kong, China; Federal University of Paraiba, BRAZIL

## Abstract

**Introduction:**

Young smokers always partake in both smoking and drinking. However, drinking undermines their likelihood to attempt quitting smoking or to successfully abstain from smoking. Hence, this trial will examine the feasibility of implementing an integrated smoking cessation and alcohol intervention in young Hong Kong Chinese people. Effect sizes of the integrated intervention (II) on self-reported and biochemically validated quit rates will also be calculated.

**Methods:**

The study will be a three-arm randomized controlled trial in a convenience sample of 150 smokers aged 18–25 years with alcohol drinking. Participants will be randomized into a standard treatment (ST), II, or control arm. The ST group will receive a brief smoking cessation intervention based on the 5A (Ask, Assess, Advice, Assist, Arrange) and 5R (Relevance, Risks, Rewards, Roadblocks, Repetition) models. The II group will receive brief advice on alcohol use based on the FRAMES (Feedback, Responsibility, Advice, Menu, Empathy, Efficacy) model in addition to the brief smoking cessation intervention. Both the ST and II groups will receive booster interventions at 1-week, 1-month, 3-month, and 6-month follow-up. The control group will receive leaflets on smoking cessation and alcohol reduction. Self-reported quitters at 6-month follow-up will be invited for biochemical validation. The primary outcomes are feasibility measures. The secondary outcomes are effect size of II on self-reported and biochemically validated quit rates at 6 months relative to control and ST. Outcomes will be assessed at baseline and at 1-week, 1-month, 3-month, and 6-month follow-ups.

**Analysis:**

Descriptive statistics will be used to calculate the feasibility measures. The three arms will be compared using analysis of variance for continuous variables and chi-square test for categorical variables. Effect sizes of II for self-reported and biochemically validated quit rates at 6 months will be determined using the generalized estimating equation model.

## Trial registration

**Trial registration number:** NCT05627765.

## Introduction

Cigarette smoking is a major public health concern. It remains the single most preventable cause of death [1]. Evidence has shown that smoking damages nearly every organ in the body [1] and is associated with numerous chronic diseases [1]. Recent evidence has indicated that smoking is the cause of death in two out of three smokers, especially when smoking is initiated at a young age [2,3]. Therefore, it is paramount to actively promote smoking cessation among young smokers to reduce smoking-attributable diseases and mortality in this population.

Evidence has shown that young smokers always partake in both smoking and drinking [4,5]. A national survey in the United States showed half of the people aged 12 or older (51.1 percent) drank alcohol in the past month and 1 in 5 (21.5 percent) used a tobacco product [6]. Moreover, alcohol use has been found to be highly related to cigarette smoking [7]. A previous study indicated that smokers were 1.32 times more likely to drink than non-smokers [7]. Another study found that smoking prevalence was 75% higher among drinkers compared with non-drinkers [8]. Alcohol use has been well-recognized as a high-risk situation that precipitates cigarette cravings and relapse [8]. One study showed that drinkers were less likely to attempt quitting and to succeed in quitting compared with non-drinkers [8].

Although a considerable number of young smokers are drinkers, current smoking cessation interventions are focused only on smoking behaviors, and alcohol drinking are generally overlooked [4]. In fact, drinking combined with smoking can lead to numerous health conditions. For example, concurrent heavy use of tobacco and alcohol can have a significant impact on the development of malignancies [7,8]. Exposure to tobacco and alcohol also results in multiplicative risk for heart attack, heart failure, stroke, gastric problems, liver cirrhosis, pancreatitis and memory loss [7]. Britton et al. [9] suggested that smoking cessation interventions that target smokers with alcohol drinking is ideal because of their significant risk for negative health and poor cessation outcomes. Therefore, an intervention that combines smoking cessation and alcohol reduction will substantially improve the physical well-being of young smokers with alcohol drinking. Furthermore, such interventions will enhance abstinence because drinking undermines the likelihood of quitting smoking and increases the chance of relapse [8].

A literature search has identified some studies that investigated integrated interventions [10–13]. For example, one pilot study evaluated the acceptability of an integrated smoking cessation and drinking intervention in young adults [10]. A total of 41 young adult smokers who were regular drinkers were randomly assigned to either the standard treatment (eight individual-based counseling sessions plus 8 weeks of nicotine patch therapy) or integrated intervention. Results demonstrated that the integrated intervention was highly acceptable to young smokers. Furthermore, young smokers who received the integrated intervention reported higher biochemically validated quit rates than those who received the standard treatment. Another study was a two-arm randomized controlled trial (RCT) comparing the efficacy of a technology-based integrated smoking cessation and alcohol intervention and a smoking cessation only intervention in adolescents [11]. A total of 1471 participants were randomized into one of the interventions and results indicated that the integrated intervention exhibited no advantages of self-reported quit rates over the smoking cessation only intervention.

Although these studies addressed the effectiveness of integrated smoking cessation and alcohol interventions, they had limitations that warrant further investigation. The study by Ames et al. [10] did not use intention-to-treat to handle missing data, leading to an overestimation of the intervention effect. Despite promising findings, the results were not statistically significant because of an insufficient sample size. In the study by Haug et al. [11], participants’ smoking status was not biochemically verified, which may have led to an overestimation of intervention effectiveness because of social desirability bias. Moreover, the study did not have a control group, precluding conclusions regarding absolute intervention effectiveness. Third and perhaps most importantly, some participants did not have alcohol drinking and therefore, an integrated intervention is less relevant to these individuals, weakening the intervention effectiveness and contributing to the non-significant findings. Because of these limitations, re-examining the effectiveness of integrated smoking cessation and alcohol interventions in young smokers in a large-scale RCT is imperative. Our pilot study will be an important prerequisite to ensure feasibility of implementing such interventions in the Hong Kong Chinese context.

The study’s research questions are:

1. What is the feasibility of an integrated smoking cessation and alcohol interventions in young smokers?
2. Will the use of an integrated smoking cessation and alcohol interventions be more effective to help young smokers achieve abstinence compared with interventions that are focused on smoking cessation alone?

### Theoretical framework

The relationship between smoking and drinking can be explained by classical conditioning [14], where the unconditioned stimulus is a stimulus that triggers an unlearned natural response. A neutral stimulus is a stimulus that produces no specific response. For example, food (unconditioned stimulus) triggers salivation in dogs (natural response), but a ringing bell (neutral stimulus) does not. However, repeated exposure to a natural stimulus that is followed by unconditioned stimulus leads to the two stimuli becoming paired. Eventually, the natural stimulus becomes a conditioned stimulus that serves as a signal for the unconditioned response (e.g., the ringing bell [conditioned stimulus] triggers salivation in the dogs [conditioned response]) [14]. This theory has been applied extensively to the relationship between drinking and smoking. Specifically, the frequent pairing of alcohol and cigarettes results in alcohol serving as a conditioned stimulus that triggers a conditioned response of cigarette craving, which increases lapse and relapse [14].

Another explanation for the relationship is reduced cognitive capacity [14]. It has been postulated that alcohol consumption creates a supportive environment for smoking, whereby drinking impairs cognitive function and reduces the amount of information that can be processed. This leads to disinhibition and engagement in risky behavior, such as smoking. Moreover, because of the impairment in cognitive function, people suppress thoughts that smoking is harmful to their health. Additionally, the contextual environment that drinking takes place often involves smokers, which can increase an individual’s motivation to smoke [14].

Standard treatment based on the ‘5A’ and ‘5R’ models proposed by the World Health Organization is recommended as a common smoking cessation intervention [15–20]. This was chosen as a comparator because we wanted to determine whether II is more effective than the current smoking cessation practice. Additionally, usual care, i.e., leaflet related to smoking cessation and alcohol drinking, was also chosen as another comparator because it only has minimal effect on smoking cessation outcomes [21]. This could maximize the comparison to show the intervention effect of II. We hypothesize that an integrated smoking cessation and alcohol intervention that aims to cease both behaviors will be more effective in helping young smokers to achieve self-reported and biochemically validated abstinence at 6 months compared with the standard treatment based on the ‘5A’ and ‘5R’ models and the control that is receiving leaflets related to smoking cessation and alcohol drinking. This hypnosis is based on our reasoning that the II will remove smokers from drinking environments that support smoking and eliminate smoking cues. As such, we will conduct a 3-arm (1:1:1), superior, parallel, non-blind, randomized controlled trial to test the feasibility and preliminary effectiveness of integrated smoking cessation and alcohol interventions in young Hong Kong Chinese smokers.

## Materials and methods

### Study design and objectives

**Fig 1:**
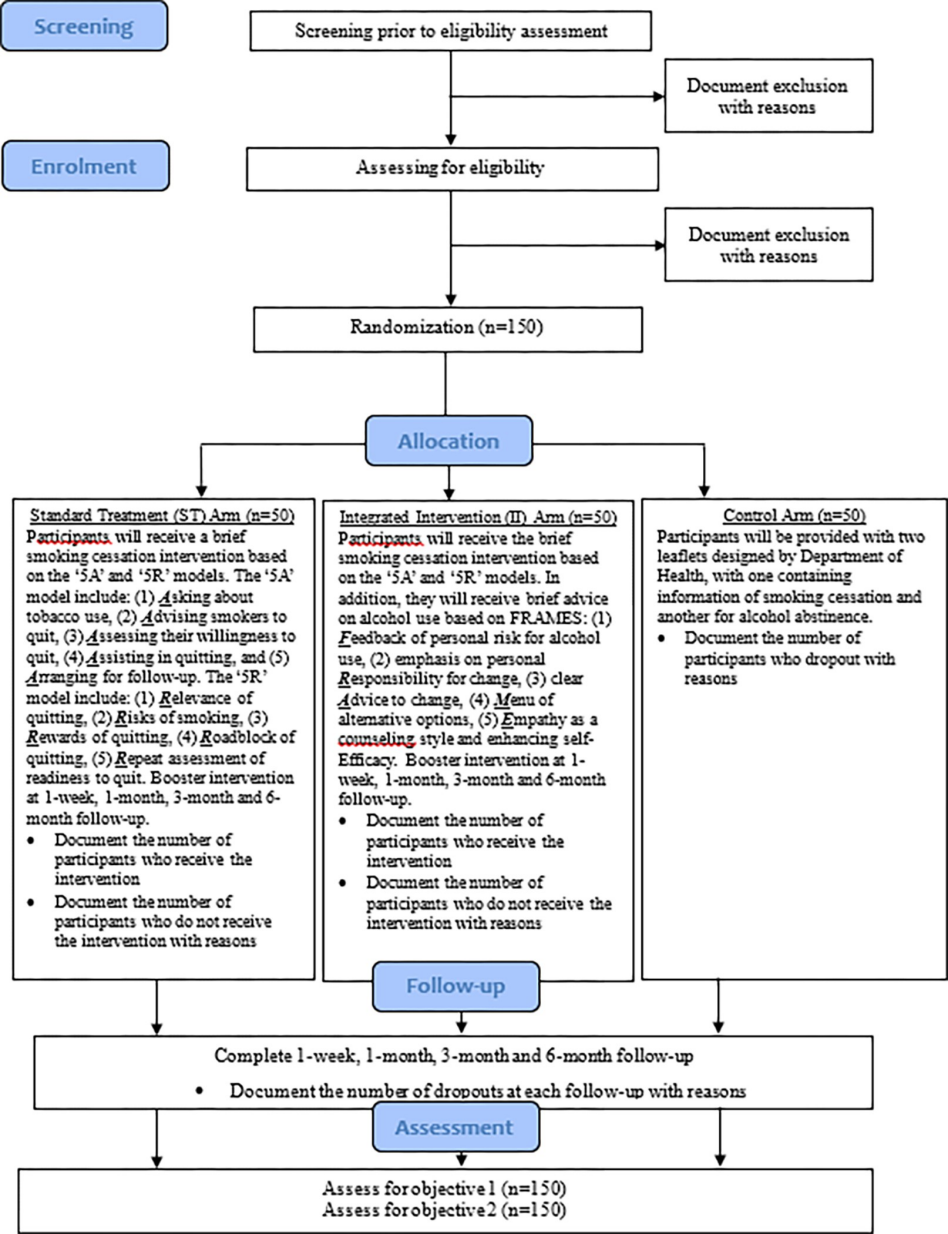
CONSORT diagram.

We will conduct a three-arm RCT to examine the feasibility of implementing an integrated smoking cessation and alcohol reduction intervention in young Hong Kong Chinese smokers. The three arms will be standard treatment (ST), integrated smoking cessation and alcohol intervention (II), and control. We will assess the feasibility of the intervention based on screening rate, eligibility rate, recruitment rate, randomization rate, attendance rate of intervention, adherence to intervention protocol, retention rate, completion rate, missing data, and adverse events. In addition, we will calculate the effect size of II based on biochemically validated and self-reported quit rates at 6 months. According to previous literature [10,11], our intervention is expected to achieve at least a small effect size relative to ST and control. Fig 1 shows the CONSORT diagram.

### Sample

Individuals are eligible for the study if they are (1) aged 18 to 25 years, (2) smoked at least one cigarette in the past 30 days, (3) consumed at least one cup of alcohol (10 ounces of beer, 4 ounces of wine, or 1 ounce of liquor) [4] in the past 30 days, and (4) speak Cantonese. Exclusion criteria include: (1) have cognition and/or communication problems, (2) have clinically reported mental disorders (anxiety, depression, etc.), (3) current use of nicotine containing medication, (4) participating in other smoking cessation interventions including pharmacological interventions. We will target participants aged 18–25 years because this age range is considered young adulthood [22]. Smokers aged <18 may also have alcohol drinking; however, their psychosocial development differs from those who reach the age of majority [23]. Therefore, we only targeted those aged 18–25 years to ensure the homogeneity. As this study aims to explore the feasibility and preliminary effect of II on smoking cessation among young smokers, we do not set any restriction on nicotine dependency. Hence, light and heavy smokers will be included. This variable will also be controlled in our GEE model. However, non-smokers will not be included. Similarly, we will not set any restriction on the participants’ alcohol dependency. However, participants will be categorized as heavy drinkers when exceeding the following threshold: drinking on a maximum of 5 days per week, maximum 3 glasses per day and 14 per week for women, and maximum five glasses per day and 21 per week for men [24]. This variable will also be controlled in the GEE model. According to Department of Health, Hong Kong [25], regular drinkers are defined as those who drink at least once in 7 days, whereas occasional drinkers are those who drink once in 30 days. We will include occasional drinkers because occasional drinking can also lead to smoking lapses and relapses [4].

There is no fixed rule for calculating sample size for a pilot study [26]. Based on the sample size estimation guide for pilot studies outlined by Birkett and Day [27]. 50 participants per arm will provide 80% power to detect differences between the groups, assuming a 5% alpha error and a 10% attrition rate. Hence, a total of 150 participants will be recruited for this study.

### Procedures

**Fig 2:**
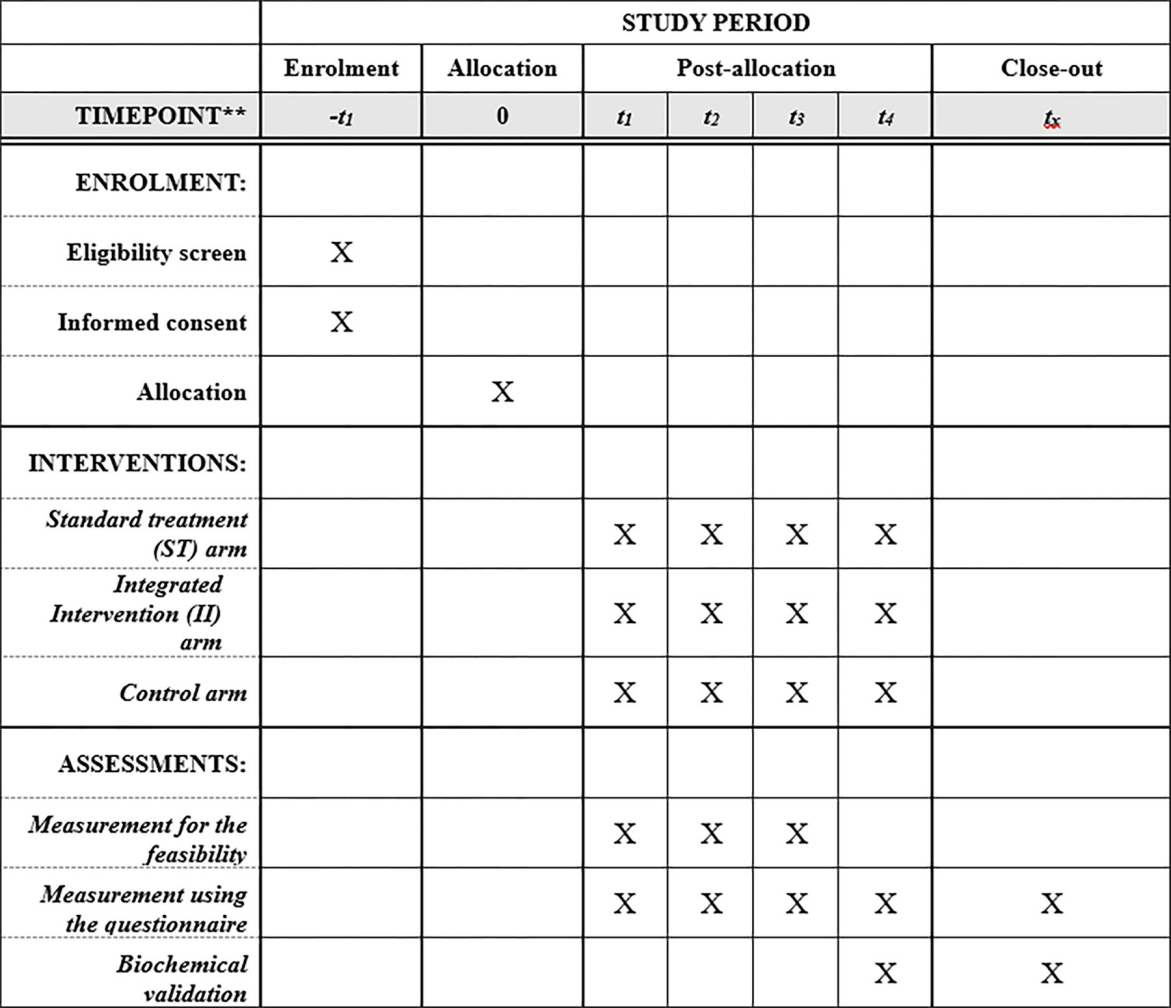
SPIRIT figure as recommended by 2013 SPIRIT statement.

This protocol is in accordance with the 2013 Standard Protocol Items: Recommendations for Interventional Trials (SPIRIT) Statement (Fig 2) and the SPIRIT checklist can be found as S1 File.

### Intervention

Standard treatment (ST) arm

Participants will receive a brief smoking cessation intervention based on the ‘5A’ and ‘5R’ models proposed by the World Health Organization. The ‘5A’ model emphasizes five steps: ***A***sking about tobacco use, ***A***dvising smokers to quit, ***A***ssessing their willingness to quit, ***A***ssisting in quitting, and ***A***rranging for follow-up [8]. Accordingly, our trained research assistant (RA) will ask participants about their tobacco use. The RA will then advise them to quit by a strong message: ‘*According to the World Health Organization*, *smoking kills one in every two smokers*. *Quitting smoking is important to protect your health*. *We are here to help you*.*’* The RA will then assess their willingness to quit by asking two questions: *‘Would you like to quit smoking*?*’* and *‘Do you think you can quit smoking successfully*?*’* If the participant replies *‘yes’* to the first question and either *‘yes’* or *‘unsure’* to the second question, they will be considered smokers who are ready to quit, and the RA will assist them to quit using the **STAR** method: ***S***et a quit date within 1 week, ***T***ell people around them about the participant quitting and solicit support, ***A***nticipate challenges to quit attempt, and ***R***emove all tobacco products. Participants who reply either *‘unsure’* or *‘no’* to the first question and *‘no’* to the second question will be considered smokers who are not yet ready to quit and the ‘5R’ model will be applied. This model comprises five components: ***R***elevance, ***R***isks, ***R***ewards, ***R***oadblocks and ***R***epetition. Accordingly, the RA will ask participants to think about how quitting smoking is personally relevant and encourage them to identify the potential risks of smoking and potential rewards of quitting. The RA will then guide participants to think about any roadblocks to quitting and provide corresponding solutions. Finally, the RA will repeat the assessment of their willingness to quit. Lastly, the RA will arrange follow-up with the participants by phone.

Booster intervention

Booster intervention will be given over the phone at 1-week, 1-month, 3-month, and 6-month follow-up, which are the crucial timepoints for booster interventions for smoking cessation [15–20]. During the conversations, the RA will ask participants about their tobacco use. If participants stopped smoking in the past 7 days, the RA will congratulate them on their success and encourage them to continue. If participants are unsuccessful at quitting, the RA will say, *‘Don’t feel disappointed*. *Would you mind telling me the reasons why you continue to smoke*?*’* The RA will provide brief suggestions to participants based on their responses and reinforce the message that quitting is beneficial for their health.

The brief smoking cessation intervention based on the ‘5A’ and ‘5R’ models is standard practice [8] and has been applied frequently in smoking cessation services [15–20].

According to the toolkit for delivering the 5A and 5R brief smoking cessation intervention developed by the World Health Organization [18], total contact time during the intervention, including follow-up, is less than 30 minutes, which is classified as a brief intervention.

#### Integrated Intervention (II) arm

In addition to the brief smoking cessation intervention based on the ‘5A’ and ‘5R’ models, participants will receive brief advice on alcohol use based on the **FRAMES** model, which is a well-established and effective brief intervention for drinking [28]. The acronym is defined as ***F***eedback of personal risk for alcohol use, emphasis on personal ***R***esponsibility for change, clear ***A***dvice to change, ***M***enu of alternative options, ***E***mpathy as a counseling style, and enhancing self-***E***fficacy.

The RA will invite participants to complete the Alcohol Use Disorders Identification Test (AUDIT) and provide personalized feedback accordingly. AUDIT is a simple instrument that was developed by the World Health Organization to identify individuals who are hazardous drinkers or have active alcohol use disorders. In Hong Kong, there is currently no recommended level of alcohol use because the harmful effects of drinking are considered to outweigh any potential health benefits [29]. A longitudinal study of population cohorts from 1990–2016 across 195 countries and territories also concluded that there is no safe level of alcohol consumption [30]. Therefore, the RA will advise participants to achieve zero alcohol consumption, provided they are classified to have low risk according to the AUDIT (scoring 1–7). After providing the personalized feedback stipulated in AUDIT, the RA will emphasize personal responsibility for change by advising, *‘No one can make this choice for you*. *It is really up to you to make a change*.*’* Additionally, the RA will advise participants, ‘*As a trained counsellor*, *I strongly advise you to stop drinking*. *This can benefit your health and increase your chance of smoking abstinence*.*’* If participants agree to stop drinking, the RA will assist them in identifying five strategies for change, e.g. alternating alcoholic drinks with soft drinks. Throughout the process, the RA will establish an empathetic relationship with participants. Moreover, the RA will support participants’ self-efficacy by saying, *‘Many people can successfully stop drinking*. *With appropriate information and our support*, *I am sure that you can do it as well*.*’* The brief advice on alcohol use will take ≤15 minutes to avoid significantly lengthening the intervention [28].

Booster intervention

Participants will receive booster interventions at 1-week, 1-month, 3-month, and 6-month follow-up. In addition to the cessation advice that will be the same as that received by participants in the ST arm, the RA will ask participants about their alcohol use. If participant stopped drinking in the past 7 days (or 30 days for 1-, 3-, and 6-month follow-up), the RA will congratulate them on their success and encourage them to continue. If participants are unsuccessful, the RA will say, *‘Don’t feel disappointed*. *Would you mind telling me the reasons why you continue to drink*?*’* The RA will provide brief suggestions to participants based on their responses and reinforce the message that stopping drinking is good for their health and will help with smoking cessation.

#### Control arm

Participants will be provided with two leaflets designed by Department of Health, Hong Kong, with one for smoking cessation and another for alcohol reduction, and scheduled telephone follow-up similar to the ST group. They will receive the placebo intervention and boosters of the same duration, promoting their fruit and vegetable intake. According to Li et al. [20], this placebo intervention and boosters do not have any intervention effect on the smoking-related outcomes.

### Intervention integrity

Firstly, all interventions will be delivered by the same RA. Secondly, the RA will be a psychology/social science graduate who is equipped with basic counseling skills. Thirdly, the RA will receive a 2-day training workshop delivered by the research team, and pass both written and oral assessments before implementing the intervention. Fourthly, 10% of delivered interventions will be randomly chosen for audio recording. The research team will listen to the audiotapes to ensure that the RA adhered to the protocol. Fifthly, weekly research meetings will be held to monitor progress.

### Outcome

The primary outcomes are feasibility measures: screening rate, eligibility rate, consent rate, randomization rate, attendance rate of intervention, adherence to intervention protocol, retention rate, completion rate, missing data, and adverse events. The secondary outcomes are effect size of II on self-reported and biochemically validated quit rates at 6 months relative to control and ST. These quit rates are the most useful outcomes to show the effectiveness of smoking cessation interventions [21]. Six months was chosen because it is the gold standard follow-up duration for evaluation in smoking cessation trials [4,15–20,31].

### Measurements

Feasibility measures include screening rate (the number of eligible smokers divided by the number of screened people), eligibility rate (the number of eligible smokers divided by the number of screened people), consent rate (the number of eligible smokers who agree to participate divided by the number of eligible smokers), randomization rate (the number of participants who are randomized divided by the number of eligible smokers who consent to participate), attendance rate(the number of participants who complete the intervention divided by the number of participants who are randomized into the treatment arms (ST and II)), adherence to intervention (the number of participants in the two treatment arms who practice the skills learned during the intervention divided by the number of participants in the two treatment arms), retention rate (the number of participants who remain in the study divided by the number of participants randomized), completion rate (the number of participants who complete the questionnaire divided by the number of questionnaires distributed), missing data (the percentage of missing data), and adverse events (unfavorable or unintended events during the study period that were not present at baseline or appear to have worsened since baseline [32]).

The proposed secondary trial outcome is effect size of II on self-reported and biochemically validated quit rates at 6 months relative to control and ST. The self-reported quit rate will be measured using a structured questionnaire designed by our team. The questionnaire will cover six areas: smoking profile (i.e., number of cigarettes smoked per day, age when starting to smoke, years of smoking, and the Fagerstrom test, which is the gold standard test for physical dependence on nicotine) [15–20,31], cessation history (i.e., previous quit attempts, when previous quit attempt was initiated, and duration of abstinence), readiness to quit, self-efficacy to quit, alcohol use, and demographics. Besides the items measuring the fact of participants, which are not possible to have the psychometric properties, the construct reliability coefficient for the Fagerstom test was 0.74 [33]. and the Cronbach’s alpha for measuring alcohol use using the Alcohol Abstinence Self-Efficacy Scale (AASE) was 0.98 [34]. The biochemically validated quit rate will be confirmed by measuring the level of carbon monoxide in expired air (< 4 ppm) and level of cotinine in saliva (< 115 ng/mL) [15–20,31].

### Randomization, blinding and concealment

We will utilize randomization to assign participants to the three groups. A RA, independent from the recruitment and intervention process, will open sequentially-numbered, opaque sealed envelopes, each containing a card indicating the allocated group. The random numbers used for group assignment will be generated in advance by another RA using a personal computer, ensuring allocation concealment. Modifying allocated interventions will not be allowed. However, the participants’ participation will be totally voluntary. They can withdraw at any time without penalty. Also, the participants can withdraw if they report any adverse event, e.g., psychological discomfort. Because smoking cessation is not part of routine care, and most instruments use self-report, blinding is not possible.

### Contamination

Because subject recruitment will be conducted in various smoking hotspots at different times, participants will be unlikely to know each other to exchange information. The possibility of contamination is low.

### Data collection

Participants will be recruited from smoking hotspots in Mong Kok. Smoking hotspots are places where many smokers congregate [31]. Mong Kok is one of the most popular places among Hong Kong youth, including young smokers [35]. The RA will approach smokers who smoke in selected smoking hotspots to deliver leaflets containing information of the study. If smokers are willing to participate, the RA will check their eligibility. Eligible smokers will be invited to join. For smokers who are ineligible or refuse participation, the RA will remind them of the health effects of smoking and drinking and refer them to the government’s quit line. The timeline of this study was presented in S2 File.

Data will be collected at baseline, 1 week, 1 month, 3 months, and 6 months, which are data collection timepoints that are commonly used in smoking cessation trials [4,19,20,31]. All participants will complete a structured questionnaire at each timepoints via telephone. Self-reported quitters at 6 months will be invited for biochemical validation.

Participants in the II arm with high (80%–100%) and low (0%–20%) adherence will be purposively selected for one-to-one, 20–30 minutes, semi-structured interviews to further assess the feasibility of II. All interviews will be conducted by the RA in a meeting room of a local university following a semi-structured interview guide and will be audio recorded. Sample size will be determined by data saturation. Based on previous smoking cessation studies [4,16], we expect data saturation to occur after interviewing 30 participants: 15 from the high adherence group and 15 from the low adherence group. As one outcome of this study is to test the feasibility of the II, no incentive will be provided to the participants.

The audit will be conducted by one auditor who was blinded to experimental or control sites. She will regularly audit the overall quality and completeness of the data, examine source documents, interview investigators and coordinators, and confirm that the clinical center has complied with the requirements of the protocol.

### Data analysis

The first objective, examining the feasibility of the II will primarily be determined by quantitative data. Qualitative data will be used to further explain the intervention feasibility. Quantitative data will be analyzed using SPSS. Descriptive statistics, i.e. frequency and percentage will be used to report the feasibility measures, including screening rate, eligibility rate, consent rate, randomization rate, attendance rate, adherence to the intervention, retention rate, completion rate, missing data and adverse events. Qualitative data will be analyzed by the traditional text analysis method using content analysis. We will use double data entry and manual checking of frequencies during data cleaning to ensure the data quality. The semi-structured interviews will be immediately transcribed and returned to informants to check for accuracy. Two team members will read the transcripts to obtain a general sense of the interviews and separately identify important statements, which will be merged into themes and subthemes. Discrepancies during analyses will be resolved in research meetings. Findings will be returned to informants for comment. Qualitative results will be reported according to Consolidated Criteria for Reporting Qualitative Research. A coding frame with quotations will be used to present results.

For the second objective, calculating the effect size of II, we will use quantitative data collected by the structured questionnaire. The three arms will be compared using analysis of variance for continuous variables and chi-square test for categorical variables. When there are violations of the normality/Gaussian assumptions in the data, alternative methods including non-parametric tests such as Kruskal-Wallis test, transformation of data and robust methods will be considered to assess the three arms, instead of ANOVA. Furthermore intention-to-treat will be applied. Participants lost to follow-up will be regarded as smokers who have the same cigarette consumption at baseline. Effect sizes of II for self-reported and biochemically validated quit rates at 6 months will be determined using the generalized estimating equation (GEE) model after controlling for baseline characteristics, relative to ST and control groups. The GEE model will be built (based on the goodness of fit) after checking for multicollinearity among the identified variables. We will apply the principle of intention to treat. Missing data will be handled by multiple imputations. The data monitoring committee (DMC) will not be set up for this study, as this trial does not involve significant safety concerns [36]. No interim analyses for efficacy will be performed during the study.

### Ethical and dissemination

Ethical approval (ref. HSEARS20211124001) has been obtained from the institutional review board of Hong Kong Polytechnic University on 28 February 2022. This study has also been registered at ClinicalTrials.gov (NCT05627765). Information sheet will be provided before seeking written consent. The children’s and parents’ participation will be totally voluntary, and withdrawal will be possible without penalty. Unfavorable or unintended events during the study period will be recorded [32]. We will assess their relatedness to the study in research meetings. Number and severity of adverse events will be recorded. All the participant data will be kept confidential.

### Patient and public involvement

There is no patient and public involvement.

## Discussion

This study will bridge the gap in the existing literature by determining the feasibility of implementing an integrated smoking cessation and alcohol intervention in young Hong Kong Chinese people. Based on the results of this study, a full-scale RCT will be conducted to further examine the effectiveness of this intervention in this population. If II is found to be effective and feasible in the Hong Kong Chinese context, it may lead to a significant change in the delivery of smoking cessation services across different regions and population. Currently, these services, which include those provided by Youth Quitline [37], focus only on smoking behavior and overlook other unhealthy behaviors, such as drinking. However, numerous surveys have observed that approximately one quarter of young smokers are drinkers. Furthermore, prevalence of drinking is similar in other smokers of different age groups. Previous studies have consistently demonstrated that drinking undermines smokers’ likelihood to attempt quitting smoking or to successfully abstain from smoking; moreover, drinking increases the chance of lapse and relapse. Therefore, we expect that an integrated intervention that combines both smoking cessation and alcohol use will significantly improve abstinence rates of smokers with alcohol drinking. Furthermore, given the multiplicative risks of chronic diseases due to the combined effects of smoking and drinking, simultaneously stopping these two unhealthy behaviors will significantly improve the physical health of smokers. Besides, future studies should expand to include different populations and geographical regions to validate the findings obtained in this study.

## Supporting information

S1 File SPIRIT 2013 checklist: Recommended items to address in a clinical trial protocol and related documents. (DOC)

S2 File Proposed timeline of work. (PDF)
