# Supplementary material for: An integrated smoking cessation and alcohol intervention among Hong Kong Chinese young people: Study protocol for a feasibility randomized controlled trial
Source: PLoS One. 2023 Aug 3;18(8):e0289633. doi: 10.1371/journal.pone.0289633 (PMC10399896; doi:10.1371/journal.pone.0289633)
Supplement: S2 File — (PDF) [file pone.0289633.s002.pdf]

Supporting Information file S2. Proposed Timeline of Work

<table>
<tr><td>Months of Study</td><td>1</td><td>2</td><td>3</td><td>4</td><td>5</td><td>6</td><td>7</td><td>8</td><td>9</td><td>10</td><td>11</td><td>12</td><td>13</td><td>14</td><td>15</td><td>16</td><td>17</td><td>18</td><td>19</td><td>20</td><td>21</td><td>22</td><td>23</td><td>24</td></tr>
<tr><td>Preparation</td><td bgcolor="#c0c0c0"></td><td bgcolor="#c0c0c0"></td><td></td><td></td><td></td><td></td><td></td><td></td><td></td><td></td><td></td><td></td><td></td><td></td><td></td><td></td><td></td><td></td><td></td><td></td><td></td><td></td><td></td><td></td></tr>
<tr><td>Subject recruitment</td><td></td><td></td><td bgcolor="#c0c0c0"></td><td bgcolor="#c0c0c0"></td><td bgcolor="#c0c0c0"></td><td bgcolor="#c0c0c0"></td><td bgcolor="#c0c0c0"></td><td bgcolor="#c0c0c0"></td><td></td><td></td><td></td><td></td><td></td><td></td><td></td><td></td><td></td><td></td><td></td><td></td><td></td><td></td><td></td><td></td></tr>
<tr><td>Implementation</td><td></td><td></td><td bgcolor="#c0c0c0"></td><td bgcolor="#c0c0c0"></td><td bgcolor="#c0c0c0"></td><td bgcolor="#c0c0c0"></td><td bgcolor="#c0c0c0"></td><td bgcolor="#c0c0c0"></td><td bgcolor="#c0c0c0"></td><td bgcolor="#c0c0c0"></td><td bgcolor="#c0c0c0"></td><td bgcolor="#c0c0c0"></td><td bgcolor="#c0c0c0"></td><td bgcolor="#c0c0c0"></td><td></td><td></td><td></td><td></td><td></td><td></td><td></td><td></td><td></td><td></td></tr>
<tr><td>Data collection</td><td></td><td></td><td bgcolor="#c0c0c0"></td><td bgcolor="#c0c0c0"></td><td bgcolor="#c0c0c0"></td><td bgcolor="#c0c0c0"></td><td bgcolor="#c0c0c0"></td><td bgcolor="#c0c0c0"></td><td bgcolor="#c0c0c0"></td><td bgcolor="#c0c0c0"></td><td bgcolor="#c0c0c0"></td><td bgcolor="#c0c0c0"></td><td bgcolor="#c0c0c0"></td><td bgcolor="#c0c0c0"></td><td></td><td></td><td></td><td></td><td></td><td></td><td></td><td></td><td></td><td></td></tr>
<tr><td>Semi-structured interviews</td><td></td><td></td><td></td><td></td><td></td><td></td><td></td><td></td><td></td><td></td><td></td><td></td><td></td><td></td><td bgcolor="#c0c0c0"></td><td bgcolor="#c0c0c0"></td><td bgcolor="#c0c0c0"></td><td></td><td></td><td></td><td></td><td></td><td></td><td></td></tr>
<tr><td>Data entry and checking</td><td></td><td></td><td bgcolor="#c0c0c0"></td><td bgcolor="#c0c0c0"></td><td bgcolor="#c0c0c0"></td><td bgcolor="#c0c0c0"></td><td bgcolor="#c0c0c0"></td><td bgcolor="#c0c0c0"></td><td bgcolor="#c0c0c0"></td><td bgcolor="#c0c0c0"></td><td bgcolor="#c0c0c0"></td><td bgcolor="#c0c0c0"></td><td bgcolor="#c0c0c0"></td><td bgcolor="#c0c0c0"></td><td bgcolor="#c0c0c0"></td><td bgcolor="#c0c0c0"></td><td bgcolor="#c0c0c0"></td><td bgcolor="#c0c0c0"></td><td bgcolor="#c0c0c0"></td><td bgcolor="#c0c0c0"></td><td></td><td></td><td></td><td></td></tr>
<tr><td>Data processing and quantitative and qualitative analysis</td><td></td><td></td><td></td><td></td><td></td><td></td><td></td><td></td><td></td><td></td><td></td><td></td><td></td><td></td><td></td><td></td><td></td><td></td><td></td><td></td><td bgcolor="#c0c0c0"></td><td bgcolor="#c0c0c0"></td><td bgcolor="#c0c0c0"></td><td></td></tr>
<tr><td>Preparation of report</td><td></td><td></td><td></td><td></td><td></td><td></td><td></td><td></td><td></td><td></td><td></td><td></td><td></td><td></td><td></td><td></td><td></td><td></td><td></td><td></td><td></td><td></td><td bgcolor="#c0c0c0"></td><td bgcolor="#c0c0c0"></td></tr>
</table>
